# USING CAREERS IN AGING WEEK (CIAW) TO PROMOTE INTERGENERATIONAL COMMUNITY ENGAGEMENT

**DOI:** 10.1093/geroni/igz038.2097

**Published:** 2019-11-08

**Authors:** Carrie Andreoletti

**Affiliations:** Central Connecticut State University, New Britain, Connecticut, United States

## Abstract

This presentation will discuss how we used CIAW 2017 to promote intergenerational community engagement by collaborating with our office of Continuing Education and AARP Connecticut to host “#DisruptAging – A Conversation Across Professions, Perspectives, and Generations.” Together we developed a half day program designed to build bridges among academic institutions, aging professionals, community collaborators, and consumers who are passionate about aging issues. We brought together a multi-generational audience of professors, students, professionals, and representatives of various community organizations to discuss ways to change the conversation around aging. This gave us an opportunity to highlight our gerontology programs and showcase our gerontology students, while students learned about aging-related careers, public policy initiatives, and organizations in Connecticut that support older adults and their families.

